# 

**Published:** 1860-01-01

**Authors:** 


### CONTENTS.

ART. PAGE

Psychological Quarterly Retrospect . . . . . . . i—xviii.

I. Paradoxical Psychology . . . . . . . . . . . . . . . 1

II. Hysteria in connexion with Religious Revivals . . . . . . . 24

III. The Parliamentary inquiry and popular Notions concerning the Treatment of Lunatics . . . . . . . . . . . . . . 45

IV. On Lesions of the Cutaneous Sensibility among the Insane . . . 68

V. William Cullen: a Psychological Study . . . . . . . . . . 82

VI. Instrumental Music in the Asylum of Quatre-Mares . . . . . 100

VII. The State in Lunacy in Ireland . . . . . . . . . . . . 103

VIII. Pauper Lunatics:

1. The Commissioners in Lunacy and the Poor-Law Board . 109

2. Statistics. . . . . . . . . . . . . . . . . 114

Medico-Legal Trial: Disputed Will—Plea of Mental Incapacity Skipper and Skipper *v.* Bodkin and Others . . . . . . . 118

